# PRP and Articular Cartilage: A Clinical Update

**DOI:** 10.1155/2015/542502

**Published:** 2015-05-05

**Authors:** Antonio Marmotti, Roberto Rossi, Filippo Castoldi, Eliana Roveda, Gianni Michielon, Giuseppe M. Peretti

**Affiliations:** ^1^Department of Orthopaedics and Traumatology, University of Torino, 10100 Torino, Italy; ^2^Molecular Biotechnology Center, University of Torino, 10126 Torino, Italy; ^3^Department of Biomedical Sciences for Health, University of Milan, 20133 Milan, Italy; ^4^IRCCS Istituto Ortopedico Galeazzi, 20161 Milan, Italy

## Abstract

The convincing background of the recent studies, investigating the different potentials of platelet-rich plasma, offers the clinician an appealing alternative for the treatment of cartilage lesions and osteoarthritis. Recent evidences in literature have shown that PRP may be helpful both as an adjuvant for surgical treatment of cartilage defects and as a therapeutic tool by intra-articular injection in patients affected by osteoarthritis. In this review, the authors introduce the trophic and anti-inflammatory properties of PRP and the different products of the available platelet concentrates. Then, in a complex scenario made of a great number of clinical variables, they resume the current literature on the PRP applications in cartilage surgery as well as the use of intra-articular PRP injections for the conservative treatment of cartilage degenerative lesions and osteoarthritis in humans, available as both case series and comparative studies. The result of this review confirms the fascinating biological role of PRP, although many aspects yet remain to be clarified and the use of PRP in a clinical setting has to be considered still exploratory.

## 1. Introduction

Platelets are one of the smallest structures among the circulating cells in blood; they are anucleate, therefore unable to replicate, their diameter is between 2 and 4 *μ*m, and they consist of cytoplasm and vesicles and survive no more than 10 days in circulation [1].

Yet, they are in continual “call of duty” because inside them lies one of the most powerful reservoirs of factors responsible for tissue repair. Indeed, these little “bag of molecules” are essential for the regenerative process in human. Recent literature has shown, in the platelet microvesicles and exosomes [2], the presence of prepackaged multiple growth factors (GFs) in an inactive form. The most relevant are platelet-derived growth factor (PDGF), transforming growth factor beta (TGF-beta), fibroblast growth factor (FGF), insulin-like Growth Factor 1 (IGF-1), Connective Tissue Growth Factor (CTGF), Epidermal Growth Factor (EGF), and Hepatocyte Growth Factor (HFG) [3, 4]. In platelets microvesicles, different microRNAs [5] involved in mesenchymal tissue regeneration [6–8] are also present and some of them, as microRNA-23b, has been hypothesized to be strictly involved in differentiation of MSC into chondrocytes [9] or, as miRNA210, has been already proposed as a therapeutic alternative to increase ligament healing by means of intra-articular injections in a small animal model [10].

Moreover, clear anti-inflammatory properties of platelet concentrates have been investigated as an associate effect in promoting tissue healing. This aspect could be a mainstay when dealing with articular cartilage lesions. It is known that an inflammatory response of appropriate magnitude and timing is essential for tissue repair as the majority of mesenchymal repair arise from a “controlled” inflammation. In this regard, lowering the inflammation in the synovial tissue would lead to a reduction of matrix-metalloproteinases, which are cartilage-matrix degrading enzymes [4].

The in vitro and preclinical evidences are the premises for the fascinating trophic properties of PRP [3, 11] that, with regards to articular cartilage, may be resumed in

- the presence of specific chondrogenic growth factors such as PDGF (that may stimulate proliferation and collagen synthesis), TGF-beta (that may enhance chondrocyte synthetic activity, matrix production, and cell proliferation and decreases the catabolic activity of IL-1), and FGF (that promotes different anabolic pathways);
- the chemotactic migration of mesenchymal stem cells (MSC) and human subchondral progenitor cells, by mechanisms that may involve a synergistic action of TGF-beta and FGF [12];
- the stimulation of the proliferation rate of MSC, independently from donor age [13, 14];
- the differentiation of MSC and surrounding cells toward a chondrogenic lineage [12, 15]: this effect has also been demonstrated in vitro with autologous human peripheral blood stem cells;
- the anti-inflammatory action of PRP [4, 16, 17];
- a hypothesizable antiapoptotic effect, by means of inhibition of apoptotic related factors (i.e., downregulation of programmed cell death protein 5 by IGF-1) [18].

Therefore, during the last 10 years, this convincing background of basic science studies about PRP has offered the clinician a promising opportunity of a new approach to the treatment of cartilage lesions and osteoarthritis.

In a clinical setting, autologous PRP may be defined as a platelet concentration product containing at least 200% of the peripheral blood platelet count. It can be produced essentially through 3 different methods:

- blood filtration and plateletpheresis, that allow obtaining PRP products with high concentrations of human platelets and platelet-derived growth factors and low numbers of contaminating leucocytes, but implying high costs [19];
- centrifugation by a “single-spinning” that has low costs and allows for a concentration of platelets up to 3 times that of baseline level, avoiding the presence of white cells;
- centrifugation by a “double-spinning,” from which a higher concentration of platelets (up to 8 fold the baseline level) may be achieved, together with the presence of a high content of leukocyte.

Following these basic concepts, 4 categories of final products of platelet concentrates can be identified for clinical purposes, as suggested by Dohan Ehrenfest et al. [20].

(1) Pure PRP, with a low content of leukocyte (P-PRP) or leukocyte-poor platelet-rich plasma. It is a preparation without leukocytes and with a low-density fibrin network after activation. It can be employed as liquid solution or in an activated gel form and it can be injected intra-articularly or simply positioned during the gelling phase on a skin wound. It can be prepared by plasmapheresis, but for a wide clinical application it may result unpractical. Anitua et al. [21] have proposed a method that implies a centrifugation at 580 g for 8 minutes of the extracted blood and a separation of the plasma fractions by pipetting. They have named the product Plasma Rich in Growth Factors or Preparations Rich in Growth Factors or EndoRet. The disadvantage is the manual pipetting steps that may hamper the reproducibility of the final product.

(2) Leukocyte rich PRP (L-PRP) is a product with leukocytes and with a low-density fibrin network after activation and it is composed of a greater content of platelets than that of pure PRP and a higher content of leukocytes. Similarly to P-PRP, it can be used as an activated gel or in a liquid form to be injected intra-articularly. It can be produced by automated double centrifugation systems and many commercial alternatives are available as Harvest Smart-PreP (Harvest Technologies, Plymouth, MA, USA), Biomet GPS III (Biomet Inc., Warsaw, IN, USA), Plateltex (Prague, Czech Republic), or Regen PRP (RegenLab, Le Mont-sur-Lausanne, Switzerland) [22].

In both P-PRP and L-PRP, the activated gel is formed through activation of platelets and fibrinogen (that creates a fibrin net) by different activating molecules (i.e., thrombin, CaCl_2_). Once activated, platelets deliver nearly 70% of their GFs within the first 10 minutes and within an hour most of the stored GFs have been already secreted [23]. The platelet-derived growth factors are firstly absorbed and then released by the fibrin net that behaves in the same way as the extracellular matrix does. So, in the PRP, a release kinetic of the platelet GFs can be conceived. This kinetic depends on the content of fibrin in the final product. This content varies according to the individual platelets and fibrinogen concentration and on the fibrin structure diversity created by the different procoagulant enzymes that induce the gel formation. These basic concepts allow explaining the length of action of PRP, once delivered at the lesion site. As an example, a recent study by Anitua et al. [24] showed that 70% of the stored PDGF is released from a PRP gel formed by a single slow centrifugation and CaCl_2_ activation in a period of 3 days, while 70% of VEGF, 60% of HGF, and 60% of IGF-1 in 24 hours; then, the GF release usually reaches a plateau and a slow secretion of the remaining content is completed up to 7-8 days.

A traditional activator of platelets is bovine or autologous thrombin. Concerns about the tolerability of the bovine products, along with some observations showing a fast release of GFs and some adverse effects in presence of thrombin [25], have suggested the use of different activators. Indeed, calcium chloride, batroxobin, and collagen type I may be used, the latter leading in vitro to a slower release of GFs compared to thrombin. It is also possible the use of a nonactivated PRP that may count on the activating effect of endogenous collagen (in situ activation), although the liquid state would restrict the clinical applicability. Moreover, to allow for a prolonged and sustained release of GFs, a novel approach theorize the use of common scaffolds (i.e., chitosan [26, 27]) as a carrier of PRP, and surprising results have been obtained with a prolonged release of GFs up to 20 days.

(3) Pure platelet-rich fibrin (P-PRF or PRFM, platelet rich fibrin matrix). This is obtained, firstly, by a slow centrifugation (approximately 1000 g) in a separator gel, that allows for the isolation of both the inactivated platelets and fibrinogen-containing plasma from the packed red and white cell fraction. Subsequently, a second centrifugation at high speed (approximately 3500 g) is performed in the presence of Ca (calcium chloride, CaCl_2_). CaCl_2_ initiates the clotting cascade and the precipitation of a fibrin scaffold, thus obtaining the formation of a gel containing fibrin as a stabilizer of the “platelet clot.” The end product is a platelet-rich fibrin scaffold, which is stiffer than the conventional PRP. It has a four-fold increment of platelets [28] and a low content of leukocyte and has the shape of a moldable tridimensional gel. A commercial alternative of P-PRF is named Fibrinet PRFM (Platelet- Rich Fibrin Matrix, Cascade Medical, Wayne, NJ, USA). This gel can be sutured or pressed in a defect site. It cannot be injected, due to the strongly activated gel form. Therefore, due to the high content in fibrin, this PRP formulation may release the platelet growth factors in a more extensive period, up to 7 days, with a high variability of the release kinetic. Indeed, an abundant release of the GFs has been observed within the first day and a gradual decrease of the release of the growth factors thereafter, within 2 days for VEGF and PDGF and within 7 days for EGF and FGF [29].

(4) Leukocyte- and platelet-rich fibrin (L-PRF), similar to the latter, but with high content of leukocyte. As P-PRF, L-PRF is a strongly activated gel with a high-density fibrin network and it cannot be injected. It has the form of a solid material and it can be locally applied at the lesion site. It was derived from a one-step centrifugation of blood without anticoagulant and without blood activator. A commercial product of L-PRF is the Intra-Spin L-PRF (Intra-Lock Inc., Boca Raton, FL, USA). This is a simple and inexpensive method and it allows producing large quantities of product in a very short time. It has been widely used in oral and maxillofacial surgery and, in orthopaedics, it has been proposed to facilitate the rotator cuff repair [30].

In general, a high content of leukocyte has been associated with antimicrobial activity, as studies have shown that L-PRP has a negative effect on the growth of* Staphylococcus aureus* and* Escherichia coli* in vitro [31]. A L-PRP is also associated with a greater presence of catabolic cytokines as matrix metalloproteinase-9 (MMP-9) and interleukin-1 beta (IL-1beta) [32, 33] as well as a greater number of platelets. As a consequence, a theoretical greater content of growth factors like PDGF and TGF-beta is linked to L-PRP. Nevertheless, an interesting study by Anitua et al. [34] has shown an important decrease of the availability of VEGF after 3 days of incubation and a decrease of PDGF release in L-PRP. Moreover, in a recent preclinical (rabbit) [35] study, L-PRP has been observed to cause greater inflammatory reactions and more undesirable side effects than P-PRP following the injection at the lesion site.

Therefore, for a proper clinical use, it is of primary importance to know what type of preparation will be created by a specific commercial system, to allow for the correct choice of a suitable final formulation. Nevertheless, for this purpose, the presence of leukocyte in regard to the treatment of cartilage lesions should not be viewed straightforwardly as a “foe,” but as a still debated issue. Indeed, in a recent in vitro study by Cavallo et al., a preparation of L-PRP was able to promote chondrocyte proliferation as well as a P-PRP at end term of the culture and the production of hyaluronan was greater with L-PRP compared to that of P-PRP [36]. More studies are needed to determine the best PRP formulation for the treatment of cartilage lesions and osteoarthritis in humans.

In the perspective of a translation from “bench to bedside,” these observations suggest that a clinical application of PRP for cartilage lesions and osteoarthritis has more questions than answers and a great number of variables have to be considered as:

- the interindividual differences in platelets and fibrin concentration;
- the different methods of PRP preparation [37];
- the unpredictable individual response to a specific method; intraindividual variations have been observed within the same method performed on samples taken at a different time periods [38] and also significant variations in PRP obtained from different preparation systems have been described in a single-donor model [39];
- the storage of platelets: fresh PRP seems to better preserve platelet function and GF-release compared to freeze-thawed PRP; nevertheless, the use of fresh PRP implies blood harvesting and PRP preparation every time a PRP injection has to be performed, while freeze-thawing would allow for a preservation of multiple PRP samples from the same patient;
- the possible use of homologous PRP produced by a blood bank; this alternative would reduce the bias derived from the interindividual platelet differences and would offer a single homogeneous and reproducible product available for clinical use, similarly to the blood transfusion concept; this method has been adopted in different clinical settings, in example for the cure of necrobiosis lipoidica in diabetic patients with good results [40]; recently, the use of allogenous PRP has been also proposed in literature for the treatment of cartilage defects in humans; allogenous PRP was used as a carrier for autologous culture expanded bone marrow MSC in the pilot study of Haleem et al. [41]; it may represent a potential opportunity in the field of PRP application for cartilage repair.

In such a complex clinical landscape, some promising studies have been completed in the last 10 years. Literature indicates that the evidence of a valid effect of PRP for cartilage repair is perceptible both for the treatment of cartilage lesions by means of reconstructive surgery and for the treatment of osteoarthritis by means of conservative intra-articular delivery.

## 2. PRP and Cartilage Surgery

A rationale for PRP application in cartilage surgery is suggested by in vitro and preclinical experiences [11] due to the trophic properties, the ability to help MSC to differentiate toward cartilage and bone in an appropriate environment and the anti-inflammatory capacity.

One of the first experiences came from the study of Sánchez et al. in 2003 [42]. They treated a patient with osteochondritis dissecans of the medial femoral condyle by means of arthroscopic reattachment of the loose chondral body and PRP injection between the crater and the fixed fragment. They obtained a promising clinical result demonstrated by magnetic resonance imaging (MRI).

Subsequently, PRP has been associated with the microfracture technique to improve the cartilage repair. The preclinical sheep model of Milano et al. [43] offered a convincing “proof of concept” of this intuition, advocating the use of gel rather than a liquid preparation for this specific surgical approach. In humans, this approach has been validated in a recent randomized study by Lee et al. [44]. These authors investigated the potential of PRP as an adjunct at the end of the microfracture procedure for knee cartilage defects up to 4 cm^2^ in patients older than 40 years of age. They used a L-PRP and the process of preparation did not imply the use of activator. PRP was injected in situ around the microfracture holes after removal of arthroscopic fluid from the joint, following the principle of the in situ activation. Their outcomes were convincing with regards to the clinical scores (IKDC and Lysholm) at 2 years and the second arthroscopic view at a short time follow-up (4–6 months). This was thought to be due to the double action of PRP in enhancing bone marrow MSC migration and activation and in reducing the inflammation and, subsequently, the pain at the surgical site. These results propose PRP as a promoter of healing process after microfracture. Moreover, theoretically, they allow for broadening the indication of this technique to a population older than 40 years of age, in which microfracture repair alone may become less efficient compared to younger patients.

However, PRP may also be used postoperatively with encouraging results. The recent comparative study of Manunta and Manconi [45] proposed a protocol of multiple L-PRP injections given at a short distance from the microfracture procedures. The authors obtained at 1 year similar results to that of the study of Lee, although their small number of cases did not lead to a statistical significance.

Similar promising effects were as well obtained in treating osteochondral talar lesion in the randomized, prospectively designed study of Guney et al. [46]. They used a L-PRP administered 6–24 hours postoperatively through the arthroscopic portal entry site. At a medium term follow-up (average 16 months), the authors observed better scores in the visual analogue scale (VAS) scale, in the American Orthopaedic Foot and Ankle Society (AOFAS) scoring system and in the Foot and Ankle Ability Measure (FAAM), compared to that of control cases. Thus, they sustained that an immediate postoperative PRP injection may improve the functional recovery of talar osteochondral lesions treated by microfracture technique.

PRP has been also locally applied by means of scaffolds, following the principles of the acellular one-stage cartilage repair. Several preclinical evidences have shown a positive effect of PRP in association with different materials [47]. Nevertheless the experience of the Rizzoli group has recommended caution when using PRP in association with their nanostructured three-layered scaffold made of collagen-hydroxyapatite [48]. Overall, these preclinical observations suggest that PRP seems to “make a difference” when combined to scaffold with a simple tridimensional structure, as a bilayer collagen matrix [49] or a microporous PLGA [50]. The positive influence of PRP may indeed be hampered by the presence of scaffolds with more complex configuration as the nanostructured membranes. These nanoscaffolds, per se, would already lead to an improved osteochondral repair through peculiar modalities like the chondrogenic effect of the nanoparticles of hydroxyapatite [51, 52]. With regard to that, recent clinical studies have proposed PRP in association with collagen or synthetic implants, showing a positive effect of PRP augmentation. In the first study, Dhollander et al. [53] treated patellar cartilage defects by means of microfractures with slow speed drilling. They covered the defect site with a collagen I/III membrane, manually inserted a L-PRP gel beneath the scaffold and named this technique “AMIC plus,” a modification of the original AMIC (autologous matrix-induced chondrogenesis) procedure. After a follow-up of 24 months, they obtained good results in terms of clinical outcome. They observed improvement in KOOS (Knee injury and Osteoarthritis Outcome Score), Tegner activity scale, Kujala patellofemoral score, and VAS scale. The MRI evaluation with the MOCART system showed an incomplete repair with subchondral lamina, bone changes, and intralesional osteophytes. Albeit confined in a low level of evidence, these results are encouraging both for the effect of PRP for enhancing cartilage repair and for the controversial treatment of patellar chondral lesions. In the studies of Siclari et al. [54, 55], polyglycolic acid- (PGA-) hyaluronan (HA) scaffolds were soaked by a P-PRP and used to cover femoral and tibial condyle defects previously treated with microfractures by means of K-wire drilling (as in the Pridie technique). As an adjunct, the tibial defect was stabilized by the PRP gelled by calcium gluconate and thrombin additives, while the femoral fixation was performed with small barbed polylactic acid (PLA) nail. Clinical improvement was demonstrated by the evidence of better KOOS at 12 months of follow-up, compared to that of the preoperative period. This result was stable also at 24 months. Biopsies taken from second look arthroscopies at 18–24 months of follow-up suggested that the repair tissue had some features of the hyaline articular cartilage. Even if these studies lack a control group, their results suggested a potential of PRP in association with a scaffold for one stage treatment of chondral lesions.

The association with MSC is an alternative method of applying the positive effect of PRP for the repair of cartilage defects. In this convincing perspective, the PRP may directly enhance the reparative properties of the MSC seeded at the defect. In the pilot study of Haleem et al. [41], autologous bone marrow cells, previously expanded for 4 weeks, were seeded in an allogeneic PRP. The PRP was obtained by double centrifugation and mixed with fibrin glue, made by fibrinogen and thrombin, to constitute platelet rich fibrin glue. This stable scaffold was kept in situ by a periosteal flap. The inoculum density of MSC was 2 × 10^6^ cells/cm^2^. The design of this study recalls the valiant works of Wakitani [56, 57]. Albeit the few cases treated, the clinical improvements, the macroscopic results of two second-look arthroscopies at 12 months and the magnetic resonance images make this study an interesting premise for future developments.

However, the strongest contribution to this approach came from the group of Giannini. Both talar and knee cartilage lesions were treated by a novel arthroscopic one-stage approach consisting of a scaffold loaded with bone marrow concentrate and platelet-rich fibrin gel (P-PRF). The rationale of this method is the combination of the positive effect of platelet growth factors and the synergistic action of CD34^+^ and CD34^−^ precursor cells harvested from iliac crest bone marrow. In that, the evidence in literature of the role of CD34^+^ precursor cells for cartilage repair are multiple in vitro [58], in vivo in preclinical models (rat, rabbit) [59, 60], and also in humans, as shown in the pilot work of Saw et al. [61]. Moreover, no major cell manipulation is involved in this technique and autologous elements are utilized. All these features lead to a reproducible and economic procedure with promising results, as shown by Giannini et al. for talar osteochondral lesions [62–64]. The authors distributed the bone marrow concentrate and the P-PRF either by mixing with a porcine collagen powder or by loading an esterified hyaluronic acid-derivative membrane. The composite was placed at the lesion site after debridement of cartilage fragments and pathologic subchondral bone and it was stabilized by an additional amount of platelet-rich fibrin gel. In the first case series, after 24 months of follow-up, they observed an improvement in the AOFAS score and a consistent repair tissue at MRI. Second-look arthroscopies at 24 months showed a macroscopic aspect similar to that of articular cartilage, with a histological staining positive for SAFRANIN-0 and with collagen type II in the intermediate and deep zone of the newly generated repair tissue. Better results were found in smaller lesions (less than 2 cm^2^) and in patients without previous surgery. They confirmed the stability of their clinical results at 4 years of follow-up, although a slight decline in the AOFAS scores was observed between 24 and 48 months postoperatively [64]. The authors compared also this technique with previously performed cartilage repair procedures by means of open field autologous chondrocyte implantation (ACI), using a periosteal flap as a sealing for the cells, and arthroscopic ACI, in which the same esterified hyaluronic acid-derivative membrane was used as a scaffold [63]. This comparative retrospective study showed a intriguing result: similar pattern of AOFAS improvement was observed for the three groups at the end of the follow-up (36 months) as well as comparable histological and MRI findings. This suggests that the single-stage association of bone marrow concentrate and the PRP may be as effective as other regenerative techniques like the ACI for the treatment of talar osteochondral lesions, with lower costs and less invasivity for the patients. An analogous approach was used by these authors to treat femoral condyle cartilage defects [65]. They firstly implanted the esterified hyaluronic acid-derivative membrane, soaked with bone marrow concentrate, at the previously debrided lesion site and, at the end of the procedure, they applied the P-PRF onto the scaffold. Similarly to the outcome in the ankle, they observed, after 2 years, clinical (KOOS and IKDC score) and MRI improvement, as well as a histological appearance of regenerated proteoglycan-rich matrix in the middle and deep zones of biopsies taken at 12 months. Their results were also confirmed at 3 years of follow-up in a case series [66]. Again, these observations indicate that the one-step approach with a combination of PRP and bone marrow concentrate may represent an interesting alternative among the different cartilage repair procedures. The studies concerning PRP application in cartilage surgery are summarized in Table 1.

Thus, considering the available literature, PRP seems to positively influence the cartilage repair process. Nevertheless, no clear evidence is yet available for ascertaining the exact contribution of PRP with respect to the surgical treatments performed alone. Indeed, the association of PRP with other biological treatments, as the implantation of MSC or the use of scaffold, and the lack of comparative studies have hampered the possibility to define the specific role of PRP in improving the outcomes. In the future, prospective clinical trials will be certainly helpful in determining the basic rules of using PRP in cartilage regenerative techniques.

## 3. PRP and Intra-Articular Injections for Cartilage Pathology

**Figure 1:**
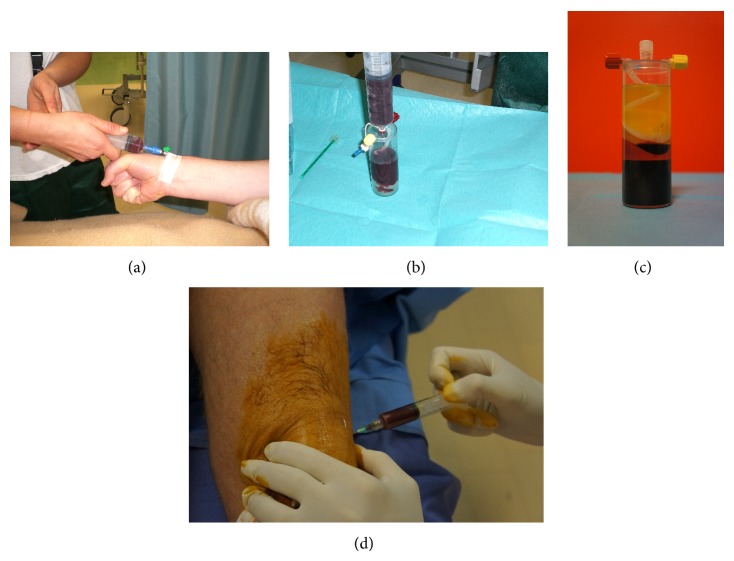
Preparation of PRP from peripheral blood sample; (a) blood aspiration; (b) transfer of patient blood into the PRP preparation chamber; (c) final PRP product; (d) intra-articular PRP injection.

The concept of a conservative treatment for cartilage degenerative lesions and osteoarthritis (OA) in humans by means of the regenerative and anti-inflammatory potential of PRP has been widely investigated. It represents an appealing approach derived from the promising in vitro and preclinical evidences [13], associated with low cost and minimally invasive procedure (Figure 1).

Several case series (Table 2) and comparative trials (Table 3) with different protocol regimens have shown a positive effect of PRP, leading to an overall improvement of the symptoms. The most common side effects reported were pain at the site of injection, lasting for some minutes, swelling and postinjective pain in the affected joint, that usually subsided in few days [67] without hampering the end-term PRP positive results.

In one of the first trials in 2010, Sampson et al. [68] used L-PRP to treat 14 patients with knee OA by means of 3 ultrasound-guided injections at 4 week intervals. They observed pain and symptoms relief at the KOOS and at the Brittberg-Peterson scores still after 12 months, although no significant differences were observed in the ultrasound measurement of the cartilage thickness during the first 6 months.

In a larger study group, Wang-Saegusa et al. [69] treated 261 patients with symptomatic OA of the knee. They used a P-PRP prepared following Anitua's technique (PRGF, “preparation rich in growth factors” or plasma-rich growth factors or platelet rich growth factors) and administered 3 intra-articular injections of autologous PRGF at 2 week intervals. Their results showed improvement after 6 months in all 4 tests used, namely, the VAS, SF-36 Health Physical parameters, WOMAC, and Lequesne Algofunctional Index.

Kon et al. [70] treated 91 patients with chondropathy or knee OA with a regimen of 3 intra-articular injections of L-PRP and follow-up of the patients for 12 months with the IKDC and EQ VAS scores. They obtained 80% of patients' satisfactions and improvement of all scores from basal values. Nevertheless, this study is the first that outlined two fundamental aspects of this approach: (i) the correlation between the worst outcomes and the older age of the patients and the advanced OA and (ii) the decrease of the clinical improvement after 6 months from the end of the treatment. They also outlined inferior outcomes in the presence of higher body mass index (BMI) and in female patients, while previous surgery did not affected the results. These findings were confirmed in a subsequent study with a longer follow-up [71]. The authors confirmed the short-term efficacy of PRP injections. They also showed that all the evaluated scores worsened from 12 to 24 months, even though a positive effect was still detectable. Indeed at 24 months clinical parameters were still higher with respect to those measured as basal values. Following these observations, the authors hypothesized a role of PRP in temporarily reducing the synovial membrane hyperplasia and modulating the cytokine level in the arthritic joint, rather than a long lasting chondroregenerative and chondroprotective effect.

Similarly to these studies, in 2012 Napolitano et al. [72] showed decrease of pain and functional improvements at 6 months at the WOMAC (Western Ontario and McMaster Universities Arthritis Index) score after 3 L-PRP injections in a group of 27 patients with knee OA (Kellgren and Lawrence 1–3) and knee cartilage disease (Outerbridge 1-2). In the same year, Torrero et al. [73] observed a similar trend of functional recovery at the VAS and KOOS scores after a single L-PRP injection in a group of 30 patients affected by knee chondropathy (Outerbridge 1–3) and Gobbi et al. [74] described improvement up to 1 year from a cycle of 2 PRP injections in 50 active patients with low to intermediate grade knee OA (Kellgren-Lawrence 1–3). In Gobbi's study, a peculiar feature was that half of the patients underwent a previous arthroscopic treatment (shaving or microfracture), but this aspect did not influence the final results after the PRP injections. More recently, in 2013, Halpern et al. [75] observed pain reduction and functional improvement at 6 months and 1 year from baseline (at the VAS and WOMAC scores) in a group of 15 patients with low grade knee OA (Kellgren-Lawrence 0–2) and they were even able to show at MRI no further cartilage degeneration at the end of the study in 73% of their patients. In the same year, Raeissadat in et al. [76] showed a beneficial clinical effect after 6 months at the SF-36 (Short Form-36) and in the WOMAC questionnaires in patients affected by knee OA ranging from low to advanced grades. Similarly to other previous studies, these authors also demonstrated an improvement in pain, stiffness, and functional capacity, with reverse relationship between patient's age and degree of pain reduction. Nevertheless, they were not able to observe any correlation between the symptoms improvement and the patients' weight, the grade of osteoarthritis or the platelet concentration (ranging in their study from 2.4 to 8.6 times). On the contrary, the study of Jang et al. [77] pointed out the negative correlation between patellofemoral joint degeneration and poorer outcomes, along with symptoms worsening after 1 year from the first injection. They also observed a relapse of pain approximately 9 months after the procedure and an adverse effect of patient's age in the final outcome.

In 2014, a study of Mangone et al. [78] again confirmed the value of L-PRP injections in a group of 79 patients selectively affected by intermediate grade knee OA (Kellgren-Lawrence 1–3). They showed improvement up to 1 year after the end of the treatment in WOMAC scale, VAS at rest, and VAS in movement. The treatment involved 3 PRP injections at 3-week interval. They outlined the role of PRP as a second approach to the treatment of knee OA due to the still high costs of this procedure compared to that of traditional HA therapy. An original observation came the same year from a study by Gobbi et al. [79]. They evaluated a group of patients after 2 years. They indicated a greater improvement of the clinical scores when the cycle of PRP injections was repeated after 1 year. However, they also perceived a decline in the performances at the end of follow-up. This was the first study supporting the value of a cyclical treatment with PRP.

PRP injections have been also applied to treat hip OA with limited results. In a preliminary study by Sánchez et al. [80], the outcomes were less satisfactory: 57% only of patients reported a clinically relevant relief of pain. An improvement in the Harris score, WOMAC pain score, and VAS scale was detected at 6 weeks but no further changes were observed.

From these case series, PRP appears to have a potential for improving knee function and quality of life of patients suffering from chondropathy or initial OA, by means of reducing the inflammation and, in a lesser extent, the degenerative articular processes. Nevertheless, some suggestions can be proposed; indeed, a better result seems to be related to

- younger patients,
- lower degree of cartilage degeneration,
- male gender,
- low BMI,
- short-term follow-up (the median duration of the efficacy of PRP injections being estimated at 9 months),
- protocols implying a repeated course of injections (i.e., after 1 year).

However, if the analysis of the case series enlightens some limits in the use of PRP injections, more promising signs come from the analysis of comparative studies regarding the benefit of PRP as a conservative therapy for chondropathy or initial OA. In fact, PRP has been matched with viscosupplementation, local anesthetics, and saline solution to assess its possible positive effect.

One of the first contributions came from the study of Sánchez et al. in 2008 [81]. The authors compared 3 injections of a single-spinning P-PRP (“preparation rich in growth factors,” PRGF) with those of a low molecular weight hyaluronic acid (LMW-HA) at a short-term follow-up. The WOMAC score showed clinical improvement: 5 weeks after the third injection significant pain reduction was recorded in 33% of PRP group while only in 10% of the control one.

Subsequently, in a complex multicenter study of Kon et al. in 2011 [82], L-PRP injections were compared with low molecular weight (LMW) and high molecular weight (HMW) hyaluronic acid (HA) administrations. At 2 months, no difference was detected between PRP and the HA, and worst results were described for patients treated with HMW-HA. Nevertheless, the authors observed at 6 months a clear positive effect of PRP, while LMW-HA treated patients showed a worsening of the performances. This effect was more evident in young patients and in the presence of early cartilage degeneration, as demonstrated by an improvement of symptoms and function at the IKCD score and at the EQ-VAS score. They did not find superior benefit of PRP with regards to HA in older patients (more than 50 years old) and in the presence of advanced OA. The patients with early OA treated with PRP displayed an “intermediate” effect, having stable score at 2 and 6 months, while the LMW-HA group worsened. Again, these results supported the hypothesis of an efficacy of PRP injections in reducing pain and symptoms and recovering articular function in patients affected by knee degeneration. This was particularly evident in patients younger than 50 years and affected by early stage of the pathology. In this subgroup of patients, these effects were longer (up to 6 months) than the well-known transient results of the HA injections. These observations were confirmed in a blinded randomized study from the same authors at 1-year follow-up [67]. They compared L-PRP injections with HMW-HA and both groups showed improvement in the performances at the clinical scores (IKDC, KOOS, EQ-VAS, Tegner), although no significant differences were observed at 12 months of follow-up. Nevertheless, a trend toward a better result was observed in the PRP group in the presence of early joint degeneration.

The positive longer effect of PRP injections was also confirmed in the randomized prospective study by Li et al. [83]. They matched PRP with sodium hyaluronate in a small group of patients with knee articular cartilage degeneration. Even if similar outcomes where observed at 4 months in both groups, significant differences in IKDC score, WOMAC score, and Lequesne index were shown at 6 months. This confirmed the more durable improvement of PRP compared to that of HA. The study of Spaková et al. [84] further strengthened these assumptions. In particular, by comparing PRP with HA, they did not notice similar early outcomes between the two groups but a constant superiority of the outcomes of patients treated by PRP injections at both 3 and 6 months of follow-up. Similarly, the study of Cerza et al. [85] in 2012 demonstrated that a formulation of P-PRP (autologous conditioned plasma, ACP) was superior to HA at any time point over 24 weeks of follow-up. The authors did not even noticed any worsening of the PRP effect in the presence of severe gonarthrosis (Kellgren-Lawrence grade III), while the outcomes of the HA control group were strongly influenced by the degree of osteoarthritis.

Conversely, in the same year, a randomized study from Sánchez et al. [86] failed to demonstrate a statistically significant superiority in overall knee performances of patients treated with P-PRP (PRGF) injections, compared to a control group treated with HMW-HA. The authors found a clear difference only in the evaluation of pain, observing a 50% decrease in the WOMAC pain subscale from baseline to 6 months in approximately 40% of patients treated with P-PRP and in 24% of patients treated with HMW-HA. Nevertheless, using the same PRP preparation (PRFG) of the study of Sánchez et al., a recent comparative study with HMW-HA has given surprising results. Indeed, Vaquerizo et al. [87] observed an improvement in both pain and physical performances (i.e., stiffness and physical function) at 24 and 48 weeks in patients affected by knee OA of Kellgren-Lawrence grades 2 to 4. Compared to the study of Sánchez et al., the main differences of the Vaquerizo's trial were (i) the HA formulation (a higher HMW-HA), (ii) the inclusion of patients with higher degree of OA, and (iii) the single HA injection compared with a cycle of 3 injections on a weekly basis of PRP. These results seem in accordance with the study of Kon et al. in 2011 with regards to HMW-HA. They support a possible biological efficacy of PRP for treatment of knee OA in a medium to long term follow-up (less than 1 year). Moreover, a recent study of Say et al. [88] showed that even a single dose of P-PRP (PRGF) may exert a benefit at the KOOS and VAS scales when compared to 3 intra-articular injections of HA in a short-term follow-up (6 months), in patients affected by bilateral gonarthrosis. This “single-shot approach” may offer the advantage of a greater safety and cost-effectiveness compared to the approach with multiple injections.

PRP has been recently compared to saline solution, as a placebo, in an interesting study by Patel et al. [89]. The authors investigated also 2 different modalities of PRP administration (1 injection versus 2 injections regimen) in patients with knee OA (Ahlbäck grade 1 and 2). In this randomized controlled trial, many of the previously reported evidences about the use of intra-articular PRP were confirmed. Indeed, the authors found that a statistically significant improvement was present both with a single dose and with 2 injections of PRP compared to placebo. They also observed that the effects were present up to 6 months of follow-up, even though at the end term evaluation the scores in the PRP groups started to deteriorate. Furthermore, better outcomes were found in patients with low grade of articular degeneration (Ahlbäck grade 1). Surprisingly, no correlation of improvement by means of PRP was found with respect to age, sex, or BMI.

A completely different approach has been used by Hart et al. [90] in a recent trial investigating the use of intra-articular PRP versus mesocain injection. The rationale of their therapeutic design was the cyclical treatment with P-PRP. In the study, patients underwent 6 injections at 1-week intervals, treatment interruption for 3 months, and then three PRP injections at 3-month intervals as a maintenance dose. This is actually the comparative study dealing with the highest number of PRP injections. The amazing result is that the outcomes of such a “PRP loaded” procedure are not so different from other “more essential” study. Indeed, at 12 months after the end of the PRP treatment, the authors found an improvement of pain and knee function at the Lysholm, Tegner, IKDC, and Cincinnati scores, but no significant influence on cartilage trophism was observed at the MRI. So, no clear benefit of such a large PRP course was validated.

Few comparative studies, finally, exist in literature regarding the use of intra-articular PRP outside the knee joint, and their results are promising. Battaglia et al. [91] investigated the effect of ultrasound-guided PRP injections versus HWM-HA intra-articular administration for the treatment of hip OA. They assessed the clinical benefit through the use of the Harris Hip Score (HHS) and the VAS scale at 1 year. They observed an improvement up to 6 months, with a slight decrease from 6 to 12 months, that paralleled the effect of the HA, and a reduction of the nonsteroidal anti-inflammatory drugs (NSAID) consumption in the PRP group in a short-term follow-up. This confirmed the temporary efficacy of PRP also for the treatment of hip OA. In a different perspective, Mei-Dan et al. [92] examined the use of PRP versus HA injections for the nonoperative treatment of talar osteochondral lesions. At a short-term follow-up (28 weeks), they found a better improvement of the Ankle-Hindfoot Scale (AHFS), and VAS scales (for pain, stiffness, and function) for the PRP-treated group, along with a similar trend for the subjective global function perceived by the patients. Again, this work confirmed the superior short-term efficacy of PRP with respect to HA also for the treatment of talar cartilage lesions.

Therefore, in the “history so far,” the results of all these comparative studies offer some basic answers to the suggestions driven by the case series in literature. Indeed, length of action, grade of OA, age of the patients, safety of the administration, and superiority of PRP towards LMW-HA and HMW-HA have been actually clarified. PRP intra-articular administration appears a relatively safe procedure with few negligible short-term side effects, as transient joint pain and effusion. There is also a strong evidence that

- PRP injection may exert a positive influence in patients affected by knee cartilage degeneration and OA (with a preliminary suggestion, even for talar osteochondral lesions);
- PRP injection may have a greater and longer efficacy than HA or placebo (saline) administration in improving pain and articular function;
- the beneficial effect of PRP injection is still temporary and it could be estimated to last up to 1 year, with a peak of action detectable at 6 months.

Moreover, the previously mentioned comparative studies agree with the recent reviews and meta-analysis from Abrams et al. [93], Anitua et al. [94], Chang et al. [95], Dold et al. [96], Khoshbin et al. [97], Pourcho et al. [98], and Tietze et al. [99], confirming the safety of PRP injections, the reduction of pain, the short term clinical benefit for symptomatic mild to moderate knee OA, and the influence of the severity of the degenerative process on the response to the treatment. Indeed, the greatest results are achieved in young patients affected by lower degree of OA or cartilage degeneration. This is in accordance with the concept that all biological therapies are positively influenced by a proper active microenvironment at the lesion site in order to reach the higher level of success.

These conclusions suggest that the primary role of PRP injections has to be found not in a direct stimulation of chondrocyte anabolic processes, but rather in a temporary modulation of joint homeostasis by the anti-inflammatory effects of PRP. This may lead to a reduction of synovial hyperplasia and cytokine production (i.e., IL-1b [17]) and, consequently, to the observed significant improvements of the clinical outcomes in a short-term follow-up.

Considering all the present evidences, literature does not justify an indiscriminate practice of PRP injections as a fist line treatment. The PRP seems to be instead an ideal candidate for a discriminative usage in the field of conservative therapy of degenerative chondropathy and mild OA, addressing specifically to a selected group of patients less than 50 years old or patients resistant to other current nonsurgical treatments [78, 95]. With regard to that, the literature continues to suggest the use of PRP injections in clinical trials [100], to allow for a deeper comprehension of the limits and the effectiveness of this therapeutic approach.

Still, in this scenario, some questions remain unsolved. It is unclear if PRP injection may sustain a substantial effect beyond the medium-term follow-up (1 year), independently from the number of doses given to the patients. Furthermore, the best administration protocol is yet to be determined, although most of the studies propose a minimal requirement of 3 doses. Solving these aspects may lead to recommend a cyclic regimen of treatment in which patients receive repetitive course of PRP injections (i.e., at 1 year interval) in order to achieve longer lasting results and to ultimately delay more invasive surgical treatments.

It is also a matter of debate if PRP may have a secondary substantial chondroprotective effect toward the progression of joint degeneration. From the current studies, no clear evidence of anabolic responses of cartilage tissue has been observed at the MRI. Nevertheless, the preclinical evidences suggest that PRP effects seem to “go beyond” a simple anti-inflammatory action. If this concept will be confirmed in the future, the clear impact of PRP on the natural history of degenerative OA will be determined.

No clear consensus exists concerning some secondary aspects of patient selection, as the influence of BMI and of sex, even though low BMI and male sex seems to lead to better clinical results.

The better activation method of PRP is unknown. Due to the differences in the study protocols available, it is impossible to determine which, if any, activating agent is preferable. However, a recent report by Textor et al. [101] has outlined that thrombin may cause inflammatory cytokine responses in joints, while no activation or the use of calcium chloride seems advisable. Moreover, novel approach, as the photoactivation [102], starts to be popular, even if the value of these new methods is still investigational.

The concurrent use of other treatment modalities is also an unclear aspect of the PRP injection approach. Indeed, PRP has been studied alone, but the combined use of PRP and other substances with dissimilar biological mechanisms may constitute a novel field of application, in order to broaden the indication and the effect of the simple PRP injection. Recently, the association of PRP and HA injections has been proposed [103] to modify the altered joint microenvironment of osteoarthritic knee. Furthermore, the percutaneous MSC injections have been recently associated with PRP in a promising trial by Pak et al. [104, 105]. They obtained MSC in the form of a stromal vascular fraction of adipose tissue. They injected a mixture composed by the cell concentrate, PRP, hyaluronic acid, and calcium chloride, obtaining promising results in patients with chondromalacia patellae. Further studies will clarify the value of this appealing therapeutic perspective.

Finally, the issue about PRP preparation method and the presence of leukocyte is a challenging topic. The controversial role of leukocyte originates from the observation that white cells may release inflammatory mediators, proteases, and reactive oxygen species in the intra-articular space, causing transient joint inflammation that may hamper the effect of PRP. From a different perspective, white cells, especially peripheral blood mononuclear cells (lymphocytes and monocytes) may exert a positive effect by means of releasing anabolic cytokines as IL-6 as well as proteins and enzymes involved in the prevention of joint infection [31, 106]. A prospective study of Filardo et al. [107] compared P-PRP (PRGF) and L-PRP in 144 patients affected by knee OA. They failed to find a significant difference in the clinical outcomes (KDC, EQ VAS and Tegner scores) up to 12 months, besides the occurrence of minor adverse events as pain and swelling in the presence of leukocytes. Nevertheless, recent in vitro evidences [108] have questioned this result pointing out the upregulation of proinflammatory factors, (IL-1beta, IL-8 and FGF-2) and the downregulation of HGF and TIMP-4 in synoviocytes in the presence of L-PRP. Therefore, the quest for the best PRP formulation is still open.

## 4. Conclusion

The review of the literature, along with the new in vitro and preclinical perspectives, gives PRP a fascinating biological possibility as a therapeutic approach for cartilage pathology. Certainly, more works has to be done in order to establish common guidelines. At this regard, high quality trials will help to clarify some of the open questions about the specific use of PRP as (i) a component of the surgical management of cartilage lesions, as well as (ii) a nonoperative injective modality for treating low grade osteoarthritis and cartilage degeneration. With regard to that, some new intuitions may be useful in the future.

In the field of surgery, a recent study outlined that the activity of PRP may be enhanced in presence of specific biphasic tridimensional porous scaffold made of collagen type I and glycosaminoglycan [109]. Indeed, this combination led to a spontaneous activation of PRP without the need of thrombin or any other activating factors and a sustained release of growth factors as PDGF, FGF-2, and TFG-beta. This might also represent an increasing evidence for the use of scaffold carrying PRP for cartilage healing.

It is, however, from the study of synergistic approaches that some attractive proposal may be suggested. A new perspective is, indeed, the modulation of PRP by means of other substances that may hasten the positive anabolic effect of PRP itself. In that, the intuitions of Jonny Huard and his research group about the use of PRP and losartan to improve muscle healing have been fundamental [110]. Indeed, the association of PRP and anti-VEGF antibody seems promising for cartilage regeneration, considering the well-known effect of VEFG in promoting hypervascularization and stimulating angiogenesis [11, 111]. The intravenous administration of a monoclonal anti-VEGF antibody (bevacizumab) has already given promising results in preclinical rabbit model [112]. Moreover, a recent preclinical study has shown the positive effect of a VEGF antagonist combined with PRP in improving cartilage repair increasing type II collagen production in a rat model [113].

The local delivery of PRP associated with systemic administration of G-CSF seems also a promising horizon. The theoretical advantage of stem cell mobilization by means of subcutaneous G-CSF injection in association with PRP administration has been recently proposed by Turajane et al. [58] in an in-vitro study. They showed that the addition of PRP and G-CSF stimulated peripheral blood stem cells to proliferate toward a chondrocyte phenotype. Furthermore, a previous study by Deng et al. have already outlined the potential of G-CSF-mobilized peripheral blood stem cells in promoting articular regeneration in OA in a preclinical rat model [59]. These observations suggest new therapeutic potential approaches for cartilage repair by means of systemic G-CSF and local PRP injection for the treatment of low-grade osteoarthritis.

So, the story “goes on” and, while new clinical studies will be proposed, preclinical evidences will emerge with new fascinating proposals: the future directions of PRP in the field of cartilage therapy is really a whole new world to be discovered.

## Figures and Tables

**Table 1:** Studies concerning PRP application in cartilage surgery.

PRP and cartilage surgery							
Authors	Year	Number of cases (age and range in y)	Study	Level of evidence	Type of PRP	Procedure and observations	Clinical results
Sánchez et al. [42]	2003	1 (age 12)	Case report	IV	/	Arthroscopic reattachment of the loose chondral body and PRP injection between the crater and the fixed fragment.	Excellent functional outcome, rapid resumption of symptom-free athletic activity.

Haleem et al. [41]	2010	5 (age 21–37)	Case series	IV	L-PRP (?)	MSC seeded in a platelet rich fibrin glue; femoral condyle cartilage lesions (size 3–12 cm^2^).	Improvement at 6 and 12 months postoperatively in Lysholm and Revised Hospital for Special Surgery Knee (RHSSK) scores.

Giannini et al. [63]	2010	25 (mean age 28 ± 9)	Retrospective comparative study	III	P-PRF	Bone marrow concentrate and P-PRF either by mixing with a porcine collagen powder or by loading a esterified hyaluronic acid-derivative membrane; talar osteochondral lesions (mean size >1.5 cm^2^).	Improvement in AOFAS score from preoperatively to 12 months and from 12 to 36 months.

Dhollander et al. [53]	2011	5 (age 24–45)	Case series	IV	L-PRP	PRP gel inserted beneath a collagen I/III membrane after the microfracture procedure; patellar focal cartilage lesions (size 1–3 cm^2^).	Improvement in VAS, KOOS and Kujala patellofemoral score at 1 and 2 years; no difference in Tegner activity scale during the 24-month follow-up.

Lee et al. [44]	2013	24 (age 40–50)	Randomized, prospectively designed study	II	L-PRP	PRP injection at the end of the microfracture procedure for femoral condyle cartilage defects up to 4 cm^2^ of size.	Better improvements in VAS and IKDC scores compared to control group at 2 years postop.

Guney et al. [46]	2013	19 (age 18–63)	Randomized, prospectively designed study	II	L-PRP	PRP injection 6–24 h after the microfracture procedure for talar osteochondral lesion (diameter less than 20 mm).	Better improvements in VAS, AOFAS, FAAM overall pain domain, and FAAM 15-min walking domain at 16 months compared to control group.

Manunta and Manconi [45]	2013	10 (age 30–55)	Randomized clinical study	II	L-PRP	3 PRP injections (1 week after surgery, then at an interval of 1 month); medial femoral condyle cartilage defects (Outerbridge II and III).	Better improvement at 6 and 12 months in IKDC score compared to control group.

Giannini et al. [62, 64]	2009, 2013	49 (mean age 28 ± 9)	Case series	IV	P-PRF	Bone marrow concentrate and P-PRF either by mixing with a porcine collagen powder or by loading a esterified hyaluronic acid-derivative membrane; talar osteochondral lesions (mean size 2 cm^2^).	Improvement in AOFAS score from preoperatively to 24 months, slight decrease at 36 and 48 months; inverse relationship at 24 months between the area of the lesion (< or >2 cm^2^) and the AOFAS score and at 48 months between the time from trauma to surgery and the AOFAS score.

Buda et al. [65, 66]	2010, 2013	20 (age 15–50)	Case series	IV	P-PRF	Hyaluronic acid membrane filled with bone-marrow concentrate; a layer of P-PRF applied onto the implanted material; femoral condyle lesions ICRS III and IV.	Improvement at 29 months postoperatively in IKDC and KOOS scores.

Siclari et al. [54, 55]	2012, 2014	52 (age 31–65)	Case series	IV	P-PRP	PGA-HA scaffolds soaked by PRP to cover the defect site previously treated by microfracture procedure; femoral and tibial condyle cartilage lesions (size 1.5–5 cm^2^).	Improvement at 12 and 24 months postoperatively in KOOS scores.

y = years; PRP = Platelet-Rich Plasma; MSC = Mesenchymal Stem Cells; P-PRP = Pure PRP, with a low content of leukocyte; L-PRP = Leukocyte rich PRP; P-PRF = Pure Platelet-Rich Fibrin.

**Table 2:** Case series concerning PRP intra-articular injections for cartilage pathology.

PRP and intra-articular injections for cartilage pathology: case series							
Authors	Year	Number of cases (age and range in y)	Study	Level of evidence	Type of PRP	Procedure and observations	Clinical results
Sampson et al. [68]	2010	14 (age 18–87)	Case series	IV	L-PRP	3 L-PRP injections at 4-week interval;	Improvement at 52 weeks from baseline in Brittberg-Peterson VAS for Pain—resting, Pain—moving, and Pain—bent knee and in KOOS for Pain and Symptom Relief.

Kon et al. [70]	2010	91 (age 24–82)	Case series	IV	L-PRP	3 L-PRP injections at 3-week interval; before the injection: Ca-chloride was added to activate platelets; knee articular damage: grades 0–4 of Kellgren-Lawrence scale.	Improvement at 6 and 12 months from baseline in IKDC, objective and subjective, and EQ VAS; tendency of worsening between 6 and 12 months.

Wang-Saegusa et al. [69]	2011	261 (mean age 48 ± 17)	Case series	IV	P-PRP	PRGF obtained following Anitua's technique; 3 injections at 2-week interval.	Improvement at 6 months from baseline in VAS pain score, Lequesne Index, SF-36 physical, WOMAC Index for pain, stiffness and functional capacity.

Filardo et al. [71]	2011	90 (age 24–82)	Case series	IV	L-PRP	3 L-PRP injections at 3-week interval; before the injection: Ca-chloride was added to activate platelets; knee articular damage: grades 0–4 of Kellgren-Lawrence scale.	Improvement at 24 months from baseline in IKDC, objective and subjective, and EQ VAS; worsening of all scores between 12 and 24 months.

Napolitano et al. [72]	2012	27 (age 18–81)	Case series	IV	L-PRP	3 infiltrations of PRP at weekly intervals; calcium gluconate 10% was added; knee articular damage: chondropathy (Outerbridge 1-2), grades 1–3 of Kellgren-Lawrence scale.	Improvement at 6 months from baseline in Numerical Rating Scale (NRS) for subjective measurement of pain and the WOMAC index for patients with knee arthritis and cartilage disease.

Gobbi et al. [74]	2012	50 (age 32–60)	Case series	IV	L-PRP	2 infiltrations of PRP at 1 month interval; articular damage: grades 1–3 of Kellgren-Lawrence scale.	Improvement at 6 and 12 months from baseline in VAS for pain, IKDC subjective and objective score, KOOS Tegner and Marx scores.

Sánchez et al. [80]	2012	40 (age 33–84)	Case series	IV	P-PRF	3 infiltrations of PRP at weekly intervals; Calcium chloride was added; hip articular damage: Tonnis 2-3.	Improvement at 6 months from baseline in VAS, WOMAC Index, and the Harris pain subscale; no significant changes in pain scores between the 6- to 7-week and 6-month time points.

Torrero et al. [73]	2012	30 (age 18–65)	Case series	IV	L-PRP	Single intra-articular injection of PRP; knee articular damage: chondropathy (Outerbridge 1–3).	Improvement at 6 months from baseline in VAS and KOOS.

Jang et al. [77]	2013	90 (age 32–85)	Case series	IV	L-PRP	PRP obtained through Magellan Autologous Platelet Separator; knee articular damage: Kellgren-Lawrence Grade 1–3.	Improvement at 6 months from baseline in VAS and IKDC score; worsening from 6 to 12 months; negative correlation with age, Kellgren-Lawrence grade and presence of patellofemoral joint degeneration.

Raeissadat et al. [76]	2013	60 (mean age 57 ± 9)	Case series	IV	L-PRP	2 L-PRP injections at 4-week interval; no exogenous activation; knee articular damage: grades 1–4 of Kellgren-Lawrence scale.	Improvement at 6 months from baseline in WOMAC Index and the native (Farsi) edition of the SF-36 questionnaire (physical and mental).

Halpern et al. [75]	2013	15 (mean age 54 age 30–70)	Case series	IV	P-PRF	1 PRP injection (Cascade system); knee articular damage: grades 0–2 of Kellgren-Lawrence scale.	Improvement at 12 months from baseline in VAS and WOMAC Index.

Gobbi et al. [79]	2014	119 (age 40–65)	Case series	IV	P-PRP	3 PRP injections at a monthly interval; articular damage: Kellgren-Lawrence Grade 1-2; 50 cases received a second cycle at the completion of 1 year.	Improvement at 24 months from baseline in VAS, KOOS, Tegner and Marx scores; tendency of worsening from 12 to 24 months; at 18 months, greater improvement in patients who received the second cycle.

Mangone et al. [78]	2014	72 (age 52–82)	Case series	IV	L-PRP	3 L-PRP injections at 3-week interval; knee articular damage: Kellgren-Lawrence Grade 2-3.	Improvement at 12 months from baseline in WOMAC index, VAS at rest, and VAS in movement; WOMAC index improved only in the first 3 months.

y = years; PRP = Platelet-Rich Plasma; P-PRP = Pure PRP, with a low content of leukocyte; L-PRP = Leukocyte rich PRP; P-PRF = Pure Platelet-Rich Fibrin.

**Table 3:** Comparative studies concerning PRP intra-articular injections for cartilage pathology.

PRP and intra-articular injections for cartilage pathology: comparative studies							
Authors	Year	Number of cases (age and range in y)	Study	Level of evidence	Type of PRP	Procedure and observations	Clinical results
Sánchez et al. [81]	2008	30 (mean age 63 ± 8)	Observational retrospective cohort study	III	P-PRP	3 PRGF injections at 1-week interval; control group: HMW-HA (30 pts); knee articular damage: Ahlbäck grades 1–4.	Better improvement of PRGF group at 5 weeks in WOMAC index.

Kon et al. [82]	2011	50 (age 30–81)	Prospective comparative study	II	L-PRP	3 L-PRP injections at 2-week interval; before the injection: Ca-chloride was added to activate platelets; control group: HMW-HA (50 pts), LMW-HA (50 pts); knee articular damage: grades 0–4 of Kellgren-Lawrence scale.	Better improvement of PRP group at 6 months in IKDC and EQ VAS scores; better results in subgroup of patients with cartilage degeneration; worsening from 2 to 6 months subgroup in patients with advanced OA; no difference between PRP and control groups in patients over 50 years.

Li et al. [83]	2011	15 (age 36–76)	Randomized prospective study	II	L-PRP	3 PRP injections at 3-week interval; before the injection: Ca-chloride was added to activate platelets; control group: sodium hyaluronate (LMW-HA) (15 pts).	No difference in IKDC score, WOMAC score, and Lequesne index between 2 groups within 4 months; better improvement of PRP group at 6 months.

Filardo et al. [67]	2012	54 (mean age 55; age 18–80)	Randomized double blind prospective comparative study	I	L-PRP	3 L-PRP injections at 1-week interval; control group: HMW-HA (55 pts); knee articular damage: grades 0–4 of Kellgren-Lawrence scale.	Improvement at 12 months from baseline in IKDC, KOOS, EQ-VAS, and Tegner for both groups; no differences between PRP group and controls; trend toward better results for the PRP group in patients with less degenerated joints.

Spaková et al. [84]	2012	60 (mean age 53 ± 12)	Prospective cohort study	II	L-PRP	3 PRP injections; control group: HA.	Better improvement of PRP group at 6 months in Numerical Rating Scale (NRS) and the WOMAC index.

Sánchez et al. [86]	2012	79 (mean age 60 ± 8)	Randomized controlled trial	I	P-PRP	3 P-PRP injections at 1-week interval; control group: HMW-HA (74 pts); knee articular damage: Ahlbäck grade I–III.	Better improvement of PRP group at 24 weeks in the percentage of patients having a 50% decrease in WOMAC pain subscale; trend toward better improvement (not significant) of PRP group in scores on the WOMAC subscales for stiffness and physical function, in Lequesne index, in the percentage of OMERACT-OARSI responders, and in the amount of acetaminophen in mg/day.

Cerza et al. [85]	2012	60 (mean age 66 ± 11)	Randomized controlled trial	I	P-PRP	4 PRP injections at 1-week interval; control group: LMW-HA (60 pts).	Better improvement of PRP group at 24 weeks in WOMAC scores; no correlation with the grade of gonarthrosis.

Mei-Dan et al. [92]	2012	15 (mean age 43 ± 18)	Randomized controlled trial	II	P-PRP	3 PRGF injections at 2-week interval; control group: HMW-HA (15 lesions; 1-week interval); ankle articular damage: Ferkel grade 1–3 osteochondral lesions.	Better improvement of PRP group at 28 weeks in AOFAS Ankle-Hindfoot Scale (AHFS), VAS for pain, stiffness, and function, subjective global function scores.

Vaquerizo et al. [87]	2013	48 (age 62 ± 7)	Randomized controlled trial	I	P-PRP	3 P-PRP injections at 1-week interval; control group: HMW-HA (48 pts; 1 single injection); knee articular damage: grades 2–4 of Kellgren-Lawrence scale.	Better improvement of PRP group at 48 weeks in WOMAC index, Lequesne index and OMERACT-OARSI responders.

Say et al. [88]	2013	45 (mean age 55)	Prospective study	II	P-PRP	1 P-PRP injections; control group: HA (45 pts; 3 injections at 1-week interval).	Better improvement of PRP group at 6 months in KOOS and VAS for pain.

Patel et al. [89]	2013	52 (group A; age 33–80) 50 (group B; age 34–70)	Randomized controlled trial	I	P-PRP	Group A (52 knees): single injection of PRP; group B (50 knees): 2 injections of PRP at 3-week interval; group C (46 knees): single injection of normal saline; knee articular damage: Ahlbäck grade I-II.	Better improvement of PRP groups at 6 months in WOMAC, VAS and overall satisfaction with the procedure; no difference between group A and B; slight worsening from 3 to 6 months.

Hart et al. [90]	2013	50 (age 31–75)	Randomized controlled trial	I	P-PRP	9 PRP injections during 1 year; control group (50 pts): 1% mesocain; knee articular damage: Grade II (fibrillation), Grade III (fissuring and fragmentation, but no bone exposed).	Better improvement of PRP groups at 12 months in Lysholm, Tegner, IKDC, and Cincinnati scores.

Battaglia et al. [91]	2013	50 (age 25–76)	Randomized controlled trial	I	L-PRP	3 PRP injections at 1-week interval; control group: HMW-HA (50 pts); hip articular damage: grades 2–4 of Kellgren-Lawrence scale.	No difference at 12 months between the groups in Harris Hip Score (HHS), NSAID consumption and VAS.

y = years; PRP = Platelet-Rich Plasma; P-PRP = Pure PRP, with a low content of leukocyte; L-PRP = Leukocyte rich PRP; P-PRF = Pure Platelet-Rich Fibrin; LMWHA = Low Molecular Weight Hyaluronic Acid; HMW-HA = High Molecular Weight Hyaluronic Acid; pts = patients; PRGF = “Preparation Rich In Growth Factors” or Plasma-Rich Growth Factors or Platelet Rich Growth Factor with a very low/absent content of leukocytes.
